# The WHO 2025 Guideline for the Prevention, Diagnosis and Treatment of Infertility: A Comprehensive Review with Focus on Male Reproductive Health

**DOI:** 10.1590/S1677-5538.IBJU.2026.0121

**Published:** 2026-03-04

**Authors:** Sandro C. Esteves

**Affiliations:** 1 Clínica de Andrologia e Reprodução Humana ANDROFERT Campinas SP Brasil ANDROFERT, Clínica de Andrologia e Reprodução Humana, Campinas, SP, Brasil; 2 Universidade Estadual de Campinas Departamento de Cirurgia Campinas SP Brasil Departamento de Cirurgia (Disciplina de Urologia), Universidade Estadual de Campinas - UNICAMP, Campinas, SP, Brasil; 3 Aarhus University Department of Clinical Medicine Aarhus Denmark Department of Clinical Medicine, Aarhus University, Aarhus, Denmark

**Keywords:** Reproductive Health, Primary Prevention, Practice Guideline [Publication Type], Review [Publication Type]

## Abstract

Infertility affects millions worldwide and is increasingly recognized as a major public-health concern. Despite advances in reproductive medicine, the lack of a unified global framework has contributed to substantial heterogeneity in clinical practice, particularly in the evaluation and management of male infertility. In 2025, the World Health Organization (WHO) issued its first comprehensive Guideline for the Prevention, Diagnosis, and Treatment of Infertility, establishing a global, evidence-based standard applicable across diverse resource settings. Notably, the guideline integrates male reproductive health throughout prevention, diagnosis, and treatment pathways, reinforcing the essential role of paternal factors in reproductive outcomes. This review summarizes the development, scope, and methodological foundations of the WHO guideline, including its use of systematic evidence synthesis, the GRADE framework, and structured consensus processes. Particular emphasis is placed on male-focused recommendations and good practice statements on lifestyle risk modification, sexually transmitted infections, standardized semen analysis, diagnostic algorithms, unexplained infertility, antioxidant supplementation, and varicocele repair. The review also clarifies the guideline's public-health scope and delineates areas that remain within the domain of specialty practice. Finally, we discuss dissemination, implementation challenges, and research priorities, highlighting persistent evidence gaps in male reproductive biology, sperm function, and clinically meaningful treatment outcomes. By aligning public-health principles with contemporary understanding of male physiology, the WHO guideline provides a global foundation for equitable and systematic infertility care.

## INTRODUCTION

Infertility affects an estimated one in six individuals worldwide and represents a growing public-health challenge with profound social, psychological, and economic consequences (1). Although substantial progress has been achieved in assisted reproductive technologies, global infertility care remains highly variable, particularly in low- and middle-resource settings where access to diagnostic services and effective treatments is limited. Historically, policy frameworks and clinical pathways have disproportionately emphasized female evaluation, reflecting entrenched assumptions about causation and societal expectations. Male infertility, in contrast, has often been under-recognized, inconsistently investigated, and insufficiently integrated into national reproductive-health strategies (2–4).

The publication of the 2025 World Health Organization (WHO) Guideline for the Prevention, Diagnosis, and Treatment of Infertility marks a significant milestone in global reproductive care (5). It is the first comprehensive WHO document to address infertility across its full spectrum, explicitly encompassing both male and female contributors. The guideline was developed to provide a globally applicable, evidence-based, and equity-oriented framework suitable for implementation across health systems with differing resource levels. Its scope extends from population-level prevention to clinical diagnosis, management, and medically assisted reproduction, with an emphasis on feasibility, person-centered care, and minimizing unnecessary interventions.

A particularly transformative aspect of the guideline is its structured integration of male infertility into the care pathway. By embedding men within prevention strategies, diagnostic evaluation, and treatment recommendations, the guideline addresses longstanding gaps that have contributed to delayed or incomplete assessment of male reproductive health. This shift is timely. Increasing evidence demonstrates that paternal health—including lifestyle factors, environmental exposures, endocrine function, and sperm molecular characteristics—plays a critical role in fertility potential, embryo development, and long-term offspring outcomes (6–13). As a result, prioritizing male reproductive assessment is not only clinically justified but essential for achieving equitable and biologically coherent infertility care (14).

This review provides a detailed examination of the WHO guideline through the lens of male reproductive health. Following a description of the guideline's rationale, scope, methodological foundations, and evidence-grading process, we analyze the male-relevant recommendations on good practice statements, prevention, diagnosis, and treatment. Particular attention is paid to lifestyle and environmental risk modification, sexually transmitted infections, semen analysis, the structure and limitations of the diagnostic algorithm, unexplained infertility, antioxidant supplementation, and varicocele management. We conclude by discussing dissemination strategies, implementation considerations, and research priorities that emerge from the guideline, highlighting key areas where evidence is currently insufficient and where future studies could strengthen subsequent guideline iterations.

## GUIDELINE OVERVIEW

Infertility has long been recognized as a significant global health concern (2, 3, 11, 15), yet until 2025, there was no unified World Health Organization guideline addressing its prevention, diagnosis, and treatment for males and females. The development of the WHO Guideline for the Prevention, Diagnosis, and Treatment of Infertility was driven by the need for a coherent, evidence-based framework to guide countries with widely differing resources, infrastructure, and clinical capacity (5). The initiative reflects WHO's broader mandate to support reproductive health as part of universal health coverage and acknowledges infertility as a condition with profound medical, psychological, and social implications for individuals, couples, and communities.

### Rationale for Developing the Guideline

The rationale for creating this guideline emerged from persistent global inequities in access to infertility care (2–4, 11, 15–17). In many regions, diagnostic evaluation is fragmented or unavailable, medically assisted reproduction (MAR) is financially inaccessible, and cultural stigma restricts help-seeking behavior—particularly for men (3, 4, 11). Prior guidance documents, explicitly focusing on male infertility, such as the American Urological Association/American Society for Reproductive Medicine (AUA/ASRM), European Association of Urology (EAU), and Brazilian Society for Human Reproduction (SBRH), provide detailed clinical recommendations but are primarily oriented toward high-resource settings (18–23). The WHO guideline was created to complement, rather than replace, such specialty resources by establishing a global baseline for essential services that can be adapted across diverse health systems. It also reflects an expanded understanding of infertility as a condition that warrants recognition and management within reproductive-rights frameworks.

Importantly, the guideline integrates male infertility throughout its structure. Historically, policy and clinical pathways have disproportionately focused on women, despite evidence that male factors contribute to infertility in up to half of all couples. The WHO document acknowledges this imbalance by embedding male evaluation within preventive strategies, diagnostic algorithms, and treatment recommendations, thereby reinforcing the principle of couple-based assessment.

### Scope and Target Audience

The scope of the WHO guideline is broad and intentionally inclusive. It covers:

- Prevention of infertility across the life course, with guidance applicable to the general population, individuals planning a pregnancy, and couples undergoing infertility evaluation.
- Diagnosis of infertility in both partners, emphasizing standardized assessment, structured history-taking, focused physical examination, and the judicious use of semen analysis.
- Treatment, including lifestyle modification, management of sexually transmitted infections (STIs), and interventions for clinical varicocele.

The guideline applies to individuals and couples attempting to conceive naturally and those who may require assisted reproduction. The document is designed to support implementation across low-, middle-, and high-resource settings, with recommendations that are feasible, equitable, and adaptable to local infrastructure and regulatory environments.

The guideline also clearly defines its boundaries. It does not attempt to provide detailed procedural guidance for ovarian stimulation, embryology laboratory techniques, endocrine management of spermatogenic dysfunction, or surgical reconstruction for obstructive azoospermia, nor does it offer guidance on genetic evaluation or advanced tests of sperm function. These omissions reflect the guideline's purpose as a public-health instrument rather than a specialty clinical manual. While it complements detailed professional-society guidelines, its primary aim is to establish a minimum global standard that can be expanded upon by national programs or specialty organizations where resources permit.

The target audience for this guideline encompasses clinicians directly involved in reproductive care—including urologists, reproductive endocrinologists, gynecologists, and primary-care providers—who must integrate its recommendations into daily practice. It also speaks to fertility nurses, midwives, counselors, and allied health professionals who deliver infertility services. Policymakers, program managers, and public health authorities are also central users, given the guideline's emphasis on systems-level implementation, resource allocation, and regulatory considerations. Researchers, educators, and trainees in reproductive medicine represent another key audience, as the guideline provides a conceptual foundation that aligns with contemporary evidence while identifying areas where data remain insufficient. By directing its content to such a diverse readership, the guideline underscores that infertility care is not confined to specialist clinics but forms an integral component of comprehensive reproductive-health systems.

## METHODS USED IN DEVELOPING THE GUIDELINE

The guideline was developed through a structured process aligned with WHO's internal standards for guideline development (Table-1). A multidisciplinary Guideline Development Group (GDG) was convened, comprising clinicians in reproductive medicine, urologists, andrologists, epidemiologists, embryologists, public health experts, methodologists, program managers, and patient representatives (5).

**Table 1:** Summary of Methods Used in Developing the WHO Infertility Guideline.

Component	Summary
**Guideline Development Group**	Multidisciplinary panel including urologists, andrologists, reproductive endocrinologists, embryologists, epidemiologists, public-health scientists, methodologists, program managers, and patient representatives.
**Formulation of Questions**	Structured using the PICO framework. Prioritized questions with global relevance, feasibility, and potential impact on health-system equity. Male-focused topics included semen analysis, repeat testing, antioxidants, varicocele, STIs, and unexplained infertility.
**Evidence Retrieval and Synthesis**	Systematic reviews commissioned or updated by WHO, complemented by targeted searches. Evidence profiles developed to summarize effectiveness, certainty, harms, acceptability, feasibility, and resource needs.
**GRADE Methodology**	Applied to assess certainty of evidence and determine strength of recommendations. Informed decisions on antioxidants (no recommendation) and varicocele repair (conditional recommendation).
**Consensus Process**	Recommendations finalized by group consensus; formal voting used when needed. Reflected balance of evidence, feasibility, values, and equity.
**External Review**	Draft guideline underwent peer review by external experts in infertility, public health, and methodology. Revisions incorporated before WHO final approval.
**WHO Approval**	Final guideline reviewed and approved by WHO's internal guideline review committee, ensuring adherence to methodological and ethical standards.

GRADE, Grading of Recommendations Assessment, Development and Evaluation; STIs, sexually transmitted infections; PICO, P stands for Patient or Problem, I is for Intervention, C is for Comparison, and O is for Outcome; WHO, World Health Organization

Clinical and public-health questions were formulated using the PICO (Population, Intervention, Comparison, Outcome) framework, prioritizing topics with high global relevance, implementation feasibility, and potential to reduce inequities in infertility care (24). Male-focused questions addressed semen analysis, indications for repeat testing, antioxidant supplementation, clinical varicocele repair, sexually transmitted infections, and criteria for unexplained infertility. Highly specialized investigations—such as advanced sperm function testing, endocrine management, and genetic evaluation—were purposely excluded because they fall outside the public health scope and are addressed in specialty guidelines.

Evidence was sourced through systematic reviews commissioned or updated by WHO, supplemented by targeted searches for randomized and observational data. Findings were synthesized into standardized evidence profiles that summarized effect size, certainty, harms, feasibility, and resource implications. The GRADE (Grading of Recommendations Assessment, Development and Evaluation) framework was applied to assess the certainty of the evidence and determine the strength of the recommendations (25). This process was central to determining, for example, that evidence for antioxidant supplementation was too heterogeneous and indirect to support a recommendation. By contrast, evidence supporting clinical varicocele repair was sufficient to justify a conditional recommendation. Recommendations were finalized through structured GDG deliberation, with formal voting and consensus. Drafts underwent external expert review before final approval by WHO's internal guideline review committee.

## SUMMARY OF GUIDELINE MALE-RELATED CONTENT

The guideline presents a framework that integrates preventive strategies, standardized diagnostic evaluation, and evidence-informed treatment options. The recommendations are organized into Good Practice Statements (GPS)—reflecting interventions supported by strong ethical, clinical, or public health principles—and formal recommendations derived from systematic evidence appraisal. The structure emphasizes global applicability, feasibility, and equity, while also incorporating clinically meaningful guidance for male infertility.

This section provides an overview of the content most relevant to male reproductive health, including the rationale and intent behind the WHO recommendations.

### Good Practice Statements

Several GPS pertain directly or indirectly to the male partner, thus reflecting interventions considered essential for quality infertility care regardless of setting (Table-2). GPS are not formal recommendations because they do not rely on GRADE-based evidence assessment; instead, they represent actions clearly supported by ethical considerations, consensus, and accumulated clinical experience.

**Table 2:** Good Practice Statements (GPS) on the General Approach and Management of Infertility in the WHO Guideline.

Good Practice Statement as written in the WHO guideline	Interpretation / Relevance to Clinical Practice
Select diagnostic tests based on the clinical findings from the medical history and physical examination to ensure that evaluation is systematic and cost-effective.	Emphasizes structured, stepwise evaluation. Diagnostic testing should follow clinical findings—not precede them—to avoid unnecessary investigations and support equitable access, especially in resource-constrained settings.
Listen to individuals and couples, respect their preferences, discuss if psychological and social or peer support is needed, and if needed, provide it or refer patients for it.	Positions infertility care within a person-centered framework. Psychological and social dimensions must be addressed alongside medical factors. Counseling and support services should be integrated or readily accessible.
Base treatment decisions on benefits and harms, patient values and preferences, feasibility, costs and availability of resources.	Reinforces shared decision-making and transparent counseling. Treatments should not be offered solely on theoretical benefit; they must be feasible, affordable, and aligned with patient priorities.
Consider the cost-effectiveness of treatment (e.g., least expensive but effective treatments should be provided initially).	Prioritizes rational, equitable sequencing of treatment. First-line options should be effective and affordable; high-cost interventions such as MAR should follow only when justified.
Discuss the plan for clinical follow-up and management of potential risks that may occur during infertility treatment.	Requires clinicians to communicate treatment expectations, safety considerations, monitoring plans, and contingency pathways. This applies to both male and female interventions.
Document the outcomes of pregnancies resulting from infertility treatment.	Supports surveillance, quality improvement, and public-health monitoring. Documentation enables outcome auditing, safety assessment, and future refinement of care pathways.

MAR = medically assisted reproduction.

Importantly, the guideline affirms that infertility care must be couple-based and that both partners should be evaluated systematically, regardless of which partner initially seeks care, a statement aligned with other relevant infertility guidelines (18–21, 26). Health-care providers are encouraged to deliver fertility information proactively, including education on modifiable risk factors, timing of intercourse relative to ovulation, and the impact of age, sexually transmitted infections, and lifestyle behaviors. The guideline also asserts that individuals and couples should have equitable access to timely, respectful, culturally appropriate infertility care. This includes counseling, psychosocial support, and linkage to specialized services when indicated. These GPSs serve as the conceptual foundation for the more detailed recommendations that follow.

### Prevention

Prevention constitutes one of the most forward-looking and transformative elements of the WHO guideline. Rather than confining infertility care to individuals who are already attempting conception, the guideline adopts a life-course, population-level perspective in which fertility awareness becomes a core component of general health promotion for both women and men. This shift is particularly significant for male reproductive health, which has historically received less structured preconception attention despite clear evidence that paternal factors influence fertility, early embryo development, and long-term offspring well-being(7, 11, 15, 27–42).

For the prevention of infertility in men, the guideline highlights four major domains, as outlined in Table-3. The guideline frames prevention as a shared responsibility between individuals, couples, and health systems. Its preventive recommendations are couple-oriented but explicitly acknowledge the need to provide men with tailored guidance. Men often underutilize primary care, seek medical attention later than women, and rarely receive structured counseling about reproductive risks. The WHO document counters this by encouraging countries to integrate fertility information into existing health-promotion platforms, such as school curricula, adolescent sexual health programs, workplace initiatives, and community outreach activities.

**Table 3:** Male-Relevant Recommendations from the 2025 WHO Infertility Guideline Relative to Prevention.

Category	Recommendation	Remarks
Information provision on fertility and infertility	For the general population of reproductive age, WHO suggests providing information about fertility and infertility using low-cost strategies or whenever there is opportunity (Conditional recommendation, very low certainty of evidence).	Low-cost strategies may include information in digital or paper format when opportunities occur in schools, at primary health care centers or at reproductive health (contraceptive, sexual health) clinics. Information adapted to local contexts and audiences, including how to reduce risk factors for infertility, lifestyle modification, age-related fertility decline/potential, and timely medical consultation, may increase the likelihood of information uptake and beneficial outcomes.
	For individuals and couples with infertility, WHO suggests providing low-cost lifestyle advice before and during infertility treatment. (Conditional recommendation, very low certainty of evidence).	Lifestyle advice may include advice to change diet, alcohol intake, smoking, physical activity and/or weight management.
Risk reduction from tobacco smoking	WHO recommends that brief advice be consistently provided by health care providers as a routine practice to all tobacco users accessing any health care settings (Strong recommendation, moderate certainty of evidence).	This is an existing WHO recommendation for the general population that also applies to individuals and couples who are planning a pregnancy, attempting to achieve a pregnancy or with infertility, given the association between infertility and current or previous history of smoking. Assessment of lifestyle, including the use of tobacco, is part of medical history when evaluating individuals and couples for infertility. Brief advice is advice to stop using tobacco – usually taking only a few minutes – given to all tobacco users, usually during a routine consultation or interaction. Brief advice should include informing individuals and couples that (i) use of tobacco, particularly smoking, is associated with a higher risk of infertility; (ii) the risk of infertility due to tobacco smoking is higher among women; and (iii) a range of interventions to assist in cessation of tobacco use exist. Brief advice should include the 5As: asking about tobacco use; advising to make a quit attempt; assessing readiness to quit; assisting in making a quit plan; and arranging a follow-up. Advice should be tailored or personalized based on individual circumstances. All adults interested in quitting smoking should be offered or referred to interventions to assist in tobacco cessation as recommended by existing WHO guidelines for preventing tobacco use uptake, promoting tobacco cessation or diagnosing and treating tobacco dependence (https://iris.who.int/handle/10665/377825).
Risk reduction from sexually transmitted infections (STIs)	Couples and individuals planning or attempting to achieve pregnancy who are accessing any health care settings should be routinely informed about sexually transmitted infections (STIs), including the risk of infertility when STIs are untreated (Good practice statement)	If symptoms of an STI are present, or if infection is confirmed, WHO guideline recommendations on the management of STIs are available (https://iris.who.int/handle/10665/342523; https://iris.who.int/handle/10665/378213)

Certainty of evidence: high (we are very confident that the true effect lies close to that of the estimate of the effect); moderate (we are moderately confident in the effect estimate: the true effect is likely to be close to the estimate of the effect, but there is a possibility that it is substantially different); low (we have limited confidence in the effect estimate: the true effect may be substantially different from the estimate of the effect); very low (we have very little confidence in the effect estimate: the true effect is likely to be substantially different from the estimate of effect). Good practice statements were made in topics where the Guideline Development Group (GDG) agreed that guidance was necessary, but a review of the evidence was not warranted because the benefits of the practice were unequivocal and other factors (such as equity) would not have an impact. Good practice statements were rooted in the fact that answers were deemed obvious by the GDG. The methodologist guided the development of good practice statements based on the Grading of Recommendations, Assessment, Development and Evaluation (GRADE) approach. Strong recommendation: For patients (most individuals in this situation would want the recommended course of action and only a small proportion would not. Formal decision aids are not likely to be needed to help individuals make decisions consistent with their values and preferences); for clinicians (most individuals should receive the recommended course of action. Adherence to this recommendation according to the guideline could be used as a quality criterion or performance indicator; for policy-makers (the recommendation can be adopted as policy in most situations); Conditional recommendation: For patients (the majority of individuals in this situation would want the suggested course of action, but many would not); for clinicians (Clinicians should recognize that different choices will be appropriate for each individual and that clinicians must help each individual arrive at a management decision consistent with the individual's values and preferences. Decision aids may be useful to help individuals make decisions consistent with their values and preferences); for policy-makers (policy-making will require substantial debate and the involvement of various stakeholders)

The guideline underscores that preventive counseling must be culturally sensitive, accessible, and matched to local realities. Men should receive clear, actionable guidance regarding the timing and frequency of intercourse, fertility at different ages, the influence of acute and chronic illnesses, and the importance of addressing genital symptoms or potential exposures early. The message is universal: male reproductive health is modifiable, and early engagement can improve fertility outcomes. Indeed, spermatogenesis is acutely sensitive to metabolic variation, oxidative stress, heat, toxins, endocrine disruptors, and genital infection. Time-to-pregnancy studies and mechanistic data indicate that paternal behaviors—even before conception—can influence embryo quality, blastulation rates, and miscarriage risk (9, 10, 16, 28, 32, 33, 35, 43–46). Integrating male fertility awareness into broader health education ensures that men receive the information necessary to understand their role in establishing optimal conditions for conception (4, 14, 15).

### Diagnosis

**Figure 1:**
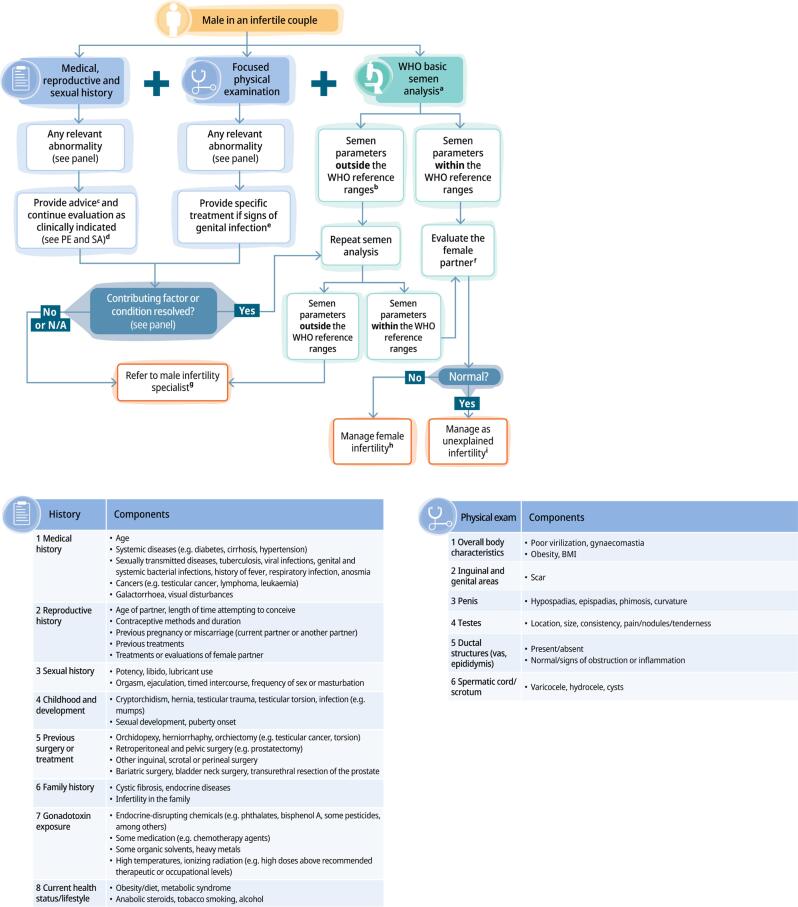
The WHO Male Diagnostic Algorithm.

^a^See the WHO laboratory manual for the examination and processing of human semen (sixth (WHO, 2021) or latest edition); ^b^Consider post-ejaculate urinalysis to rule out retrograde ejaculation if low (or no) semen ejaculate volume; see WHO laboratory manual for the examination and processing of human semen (sixth (WHO, 2021) or latest edition); ^c^See Chapter 4 in the guideline (World Health Organization, 2025), for details on information provision; ^d^Evaluation should include PE and SA regardless of history findings; ^e^See Chapter 4 in the guideline (World Health Organization, 2025) and the WHO guideline for the management of sexually transmitted infections (World Health Organization, 2021); ^f^Female evaluation is essential and should proceed regardless of semen analysis outcome; see Chapter 5 in the guideline (World Health Organization, 2025) for the assessment of the female partner; ^g^Healthcare provider with appropriate qualifications; for example, urologist, clinical andrologist or reproductive medicine specialist with relevant qualifications; ^h^See Chapters 6, 7 and 8 in the guideline (World Health Organization, 2025); ^i^See Section 5.8 and Chapter 10 in the guideline (World Health Organization, 2025). Abbreviations: N/A, not applicable; PE, physical examination; SA, semen analysis; WHO, World Health Organization. Reprinted from: Guideline for the prevention, diagnosis, and treatment of infertility. Geneva: World Health Organization; 2025. License: CC BY-NC-SA 3.0 IGO: (https://creativecommons.org/licenses/by-nc-sa/3.0/igo).

Diagnosis represents one of the most actionable components of the guideline. Diagnostic evaluation is organized around a structured, stepwise approach that integrates history, physical examination, and semen analysis, designed to ensure that every man in an infertile couple receives a basic, meaningful, and systematic assessment, regardless of geographic setting, clinician background, or health-system resources (Figure-1).

The algorithm outlines a stepwise, resource-sensitive pathway that begins with a comprehensive medical history and physical examination, followed by semen analysis in accordance with the WHO 6th edition laboratory standards. Abnormal findings prompt targeted management—such as treatment of genital infections or referral for further diagnostic steps (e.g., hormonal, genetic, or imaging evaluation)—culminating in evidence-based intervention led by male infertility specialists, typically urologists. This pragmatic framework integrates prevention, diagnosis, and referral within the broader context of equitable reproductive care.

These elements are integrated through a diagnostic algorithm that functions primarily as a triage system rather than a full clinical decision tree, enabling non-specialists to address reversible causes while promoting timely referral to clinicians with expertise in male infertility—most commonly, urologists.

The first step identifies modifiable risk factors (e.g., tobacco use, heat exposure), reversible causes (e.g., infection), and clinical abnormalities (e.g., varicocele, testicular atrophy). Standardized semen analysis should be conducted according to the WHO manual (47, 48): normal result → no repeat; abnormal result → repeat at ≥11 weeks, following the duration of one spermatogenic cycle (49). If reversible or treatable causes such as lifestyle exposures, genital infection, or medication effects are identified, these should be addressed first, followed by reassessment. Additionally, if semen parameters remain below reference limits after repeat testing, or if history/exam reveals persistent abnormalities, referral to clinicians experienced in male infertility (typically urologists) is indicated. The most relevant findings in history/exam warranting referral include palpable varicocele, endocrine or sexual dysfunction, symptoms or signs of genital tract obstruction, suspected genetic abnormalities, and complex medical conditions affecting reproduction. By contrast, the diagnosis of unexplained infertility is established only when the male partner has an unremarkable medical, reproductive, and sexual history, physical examination reveals no clinically significant abnormalities, and semen parameters fall within WHO reference ranges, and the female partner has normal ovulatory function and patent fallopian tubes.

The algorithm thus helps prevent the common situation in which male evaluation is reduced to a semen analysis alone (50, 51). By explicitly linking abnormal findings with specialist referral, the guideline reinforces the essential role of urologists in male reproductive care. Additionally, the WHO provides a detailed template for comprehensive male reproductive history-taking, covering developmental conditions, prior genital infections, systemic diseases, surgeries, medications, lifestyle exposures, occupational hazards, sexual function, previous fertility, and timing/frequency of intercourse (Figure-2- See Appendix). This structured approach helps ensure that clinically relevant risk factors are not overlooked, particularly by providers without specialized training.

### Physical Examination

The guideline recommends a focused physical examination assessing testicular size and consistency, presence of varicocele, abnormalities of the epididymis or vas deferens, signs of hypogonadism or endocrine disorders, and evidence of genital infection. By incorporating physical examination into the diagnostic algorithm, WHO stresses that male evaluation cannot be laboratory-only and must include clinically relevant observations that remain the domain of trained clinicians, particularly urologists. This examination often yields findings that meaningfully alter diagnostic direction. For example, a palpable varicocele, absent vas deferens, and signs of hypogonadism each require different diagnostic and therapeutic strategies that cannot be inferred from semen analysis alone (6, 11, 12, 19, 20, 27, 34, 52–59).

### Semen Analysis

Semen analysis remains the cornerstone laboratory test of male evaluation in the guideline. Two explicit recommendations govern its use: If semen parameters fall within WHO reference ranges, no repeat test is required. If one or more parameters fall outside the reference ranges, the test should be repeated after a minimum of 11 weeks (Table-4). The guideline stresses that semen analysis must be performed according to the latest WHO laboratory manual, which provides standardized procedures and updated reference limits (47, 48).

**Table 4:** Male-Relevant Recommendations from the 2025 WHO Infertility Guideline Relative to Diagnosis.

Category	Recommendation	Remarks
Semen analysis	For males (in couples with infertility) with one or more semen parameters outside the WHO reference ranges, WHO suggests repeating the semen analysis after a minimum of 11 weeks (Conditional recommendation, very low certainty of evidence). For males (in couples with infertility) with all semen parameters within the WHO reference ranges, WHO suggests not repeating the semen analysis (Conditional recommendation, very low certainty of evidence).	The latest edition of the WHO laboratory manual for the examination and processing of human semen provides WHO reference ranges for semen parameters and details about the standardized procedures for semen collection and analysis (https://iris.who.int/handle/10665/343208).
Diagnosis of unexplained infertility	WHO suggests making a diagnosis of unexplained infertility in a couple when all the following have occurred: (i) Failure to achieve pregnancy after 12 months of regular unprotected sexual intercourse; (ii) Normal physical examination and medical history in both the male and female; (iii) Presumptive confirmation of ovulation and patent tubes in the female partner: and (iv) Semen parameters that are within the WHO reference ranges in the male partner (Conditional recommendation, very low certainty of evidence).	NA

Certainty of evidence and strength of recommendation: see Table 3 legend. NA = not applicable; WHO = World Health Organization

The WHO approach aligns with the 2024 EAU guideline, which likewise recommends repeat testing only when the first analysis is abnormal (21). This contrasts with the AUA/ASRM guideline, which recommends at least two semen analyses by default to account for biological variability (18). The difference reflects scope: WHO aims for a globally feasible minimum standard, while specialty societies aim for improving diagnostic precision in high-resource environments.

Nevertheless, it is crucial to underscore the limitations of a basic semen analysis, focusing on volume, count, motility, and morphology (17, 60, 61). It should not be interpreted as a measure of sperm function. Although conventional parameters capture broad features of spermatogenesis, they do not reliably reflect DNA fragmentation, chromatin packaging, epigenetic marks, mitochondrial performance, and sperm-borne RNA payloads. These molecular and functional attributes can be assessed by using specialized tests, and results may influence fertilization, embryo development, blastocyst progression, and miscarriage risk (6, 35, 50, 51— 62, 63). Therefore, clinicians must interpret semen parameters within clinical contexts, recognizing that patient history, physical findings, and reproductive outcomes may diverge from what basic parameters alone imply.

## Treatment

The WHO treatment recommendations are deliberately conservative, reflecting limited evidence and the need to set minimum standards that are feasible, equitable, and implementable across health systems with vastly different resources. Only two areas yield specific male-directed recommendations: antioxidants and varicocele (Table-5). Other domains—such as hormonal therapy for idiopathic infertility, advanced sperm function testing, genetic evaluation, ejaculatory dysfunction, obstructive azoospermia, or surgical sperm retrieval—are outside the scope of this WHO guideline and are covered in detail in specialty clinical practice guidelines (6, 18–21, 26, 56). This delineation is intentional, reflecting the WHO's mandate to focus on public-health–oriented, globally applicable guidance.

**Table 5:** Male-Relevant Recommendations from the 2025 WHO Infertility Guideline Relative to Treatment.

Category	Recommendation	Remarks
Use of antioxidants	For males with infertility and one or more semen parameters outside the WHO reference ranges who are attempting to achieve pregnancy with or without medically assisted reproduction, the WHO infertility Guideline Development Group (GDG) did not make a recommendation for or against the use of antioxidant supplements.	Optimal nutrition is important during the pre-pregnancy period for the couple; however, the effects of antioxidant supplements for males with specific male-factor pathologies in couples with infertility are currently not known.
Varicocele treatment – treatment vs expectant management	For males with infertility and clinical varicocele, WHO suggests surgical or radiological treatment over expectant management (Conditional recommendation, low certainty of evidence)*.	Males with clinical varicocele and semen parameters that are outside the WHO reference ranges are more likely to benefit from receiving treatment for varicocele, compared to men with semen parameters within the WHO reference ranges.
Varicocele treatment – type of treatment	For males with infertility undergoing treatment of varicocele, WHO suggests using either surgical or radiological treatment (Conditional recommendation, very low certainty of evidence)*.	When selecting whether to use surgical or radiological treatment, consider feasibility, the availability of trained health care providers and patient preferences regarding the type of treatment procedure.
Varicocele surgery – choice of surgical method	For males with infertility undergoing surgical treatment of varicocele, WHO suggests using microscopic surgery rather than other surgical procedures (Conditional recommendation, very low certainty of evidence)*.	Subinguinal microsurgery is a common surgical varicocelectomy procedure, while other surgical procedures include non-microscopic open approaches (such as inguinal and retroperitoneal) and laparoscopic methods. In settings where the expertise to perform microscopic surgery is not available, other surgical techniques may be used.
Varicocele surgery – open approaches	For males with infertility undergoing non-microscopic surgical treatment of varicocele, WHO suggests using either inguinal or retroperitoneal surgical procedures (Conditional recommendation, very low certainty of evidence)*.	When selecting whether to use an inguinal or retroperitoneal surgical procedure, consider feasibility and the availability of trained health care providers.

Certainty of evidence and strength of recommendation: see Table 3 legend.

* This recommendation applies to males with varicocele in couples with fertility who are not undergoing treatment with assisted reproductive technology (ART).

### Antioxidant Supplementation

Oxidative stress is recognized as an important biological mechanism that can impair sperm function (64). Reactive oxygen species (ROS) influence sperm membrane integrity, motility, DNA fragmentation, chromatin compaction, and mitochondrial activity (65). Evidence from mechanistic studies strongly supports the hypothesis that excessive ROS disrupts sperm function and may impair fertilization and early embryo development (64, 65). These biological insights have motivated widespread clinical interest in oral antioxidant supplementation among infertile men.

The GDG examined evidence from a systematic review (66) and targeted search for randomized controlled trials up to April 2024 evaluating antioxidant supplementation in men with infertility and at least one semen parameter below WHO reference limits. The evidence base showed considerable heterogeneity in type of antioxidant (e.g., vitamin C, vitamin E, L-carnitine, coenzyme Q10, selenium, zinc, N-acetylcysteine, and multi-ingredient formulations), dosing and duration, study populations, and outcome measures, with most trials focusing on surrogate outcomes (semen parameters) rather than clinically meaningful endpoints (pregnancy, live birth).

The guideline therefore issues no recommendation for or against routine antioxidant supplementation in infertile men with semen abnormalities (Table-6). This is not equivalent to stating that "antioxidants do not work," nor is it a statement of insufficient evidence. Instead, it reflects an inability to formulate a global recommendation due to the heterogeneity of the evidence and the uncertainty around clinically meaningful outcomes. Nevertheless, the guideline emphasizes that optimal nutrition remains important during the pre-pregnancy period, even though the specific effects of antioxidant supplements on fertility outcomes remain uncertain. For clinicians, this means antioxidant therapy can be discussed on a case-by-case basis with appropriate counseling on benefits and uncertainties.

**Table 6:** Male-Specific WHO Recommendations Mapped to Evidence-to-Decision (EtD) Considerations.

Male WHO Recommendation	Eth Consideration	Summary of How Eth Informed the Recommendation
Repeat semen analysis only when one or more parameters are below WHO reference ranges; do not repeat when all parameters are within reference limits	Balance of benefits and harms	Repeat testing provides confirmation when abnormal, but adds no clinical value when initial parameters are within reference limits. Avoids harm from unnecessary delay, anxiety, and cost.
	Certainty of evidence	Certainty moderate, grounded in long-standing WHO manual methodology and international laboratory experience.
	Values and preferences	Little variability—patients and clinicians generally prefer to avoid unnecessary tests.
	Acceptability	High global acceptability; aligns with the WHO manual and with EAU guidelines.
	Costs/resources	Reduces costs by minimizing unwarranted repeat testing.
	Feasibility	Highly feasible even in resource-limited settings.
	Equity	Increases equity by standardizing a low-cost approach worldwide.
	Strength (GRADE)	Conditional ("WHO suggests…") —because thresholds and feasibility vary across settings.
No recommendation for or against use of antioxidant supplements for infertile men with semen parameters below WHO reference ranges	Balance of benefits and harms	Benefits uncertain; heterogeneity in products and outcomes; potential harms (reductive stress) not well defined.
	Certainty of evidence	Low due to heterogeneity in formulations, dosing, populations, and reliance on surrogate outcomes (semen parameters rather than pregnancy/live birth).
	Values and preferences	High variability; some patients expect benefit, others are skeptical; clinicians differ widely in prescribing behavior.
	Acceptability	Varies significantly across regions; unregulated supplement markets contribute to inconsistency.
	Costs/resources	Supplements often costly and out-of-pocket; cost-effectiveness unknown.
	Feasibility	Feasible but unregulated; inconsistent product quality complicates implementation.
	Equity	Potential to worsen inequity if men spend significant resources on interventions without proven benefit.
	Strength (GRADE)	No recommendation —due to insufficient evidence for benefit or harm.
Repair of clinical varicocele in infertile men with abnormal semen parameters	Balance of benefits and harms	Benefits—improvement in semen parameters and pregnancy rates—outweigh harms when varicocele is clinical/palpable.
	Certainty of evidence	Moderate certainty (supported by trials and observational studies).
	Values and preferences	Majority of patients favor intervention in hopes of natural conception; variability low.
	Acceptability	Acceptable worldwide; microsurgical approach preferred, but alternative techniques acceptable where microsurgery not available.
	Costs/resources	Cost-effective relative to immediate use of MAR; resource needs vary by region.
	Feasibility	Globally feasible with flexibility in surgical approach; microsurgery availability influences choice.
	Equity	Improves equity by supporting a treatment that may reduce reliance on MAR.
	Strength (GRADE)	Conditional ("WHO suggests…") —because of variable surgical capacity and differences in feasibility across regions.

MAR = medically assisted reproduction; GRADE = Grading of Recommendations Assessment, Development and Evaluation

### Varicocele Repair

Varicocele remains one of the most common correctable causes of male infertility worldwide (6, 34, 39, 45, 67–83). It was the only male condition for which the WHO issued a positive treatment recommendation (Table-5), reflecting both the quality of the available evidence and the guideline's public health scope.

Under a PICO framework, clinical varicocele was found to be the primary male infertility condition for which consistent, moderate-certainty evidence demonstrates improvements in semen parameters and a probable benefit in pregnancy rates when repair is performed in appropriately selected men. Repair is suggested for men with a clinical (palpable) varicocele, infertility, and abnormal semen parameters, thus aligned with the recommendations provided by most male infertility guidelines (6, 18, 19, 21, 26). The overall certainty of evidence was rated as moderate, sufficient to support a conditional recommendation but not a strong one (Table-6).

The guideline states that microsurgical repair is preferred when available, due to lower recurrence and complication rates. However, in settings without microsurgical expertise, inguinal, retroperitoneal, or radiological approaches remain acceptable alternatives. Therefore, lack of access to microsurgery should not prevent offering varicocele repair where clinically indicated.

In contrast, evidence for subclinical varicocele remains inconclusive, with trials showing inconsistent benefit and no reproducible improvement in natural or assisted reproduction outcomes (84). The GDG therefore determined that recommending repair of subclinical varicoceles would not meet the thresholds for certainty, feasibility, or cost-effectiveness required for global adoption. Similarly, the guideline refrained from making recommendations on other male infertility interventions—such as hormonal therapy for idiopathic infertility or pre-sperm retrieval for males with non-obstructive azoospermia—because evidence quality was judged insufficient, heterogeneous, or primarily based on surrogate outcomes, which limited their suitability for global guidance.

By highlighting clinical varicocele, the WHO provides a clear, implementable, and evidence-aligned recommendation that can be applied across resource settings. This targeted approach reduces unnecessary testing and intervention, promotes appropriate use of surgical and radiological resources, and reinforces the importance of accurate physical examination as the cornerstone for identifying clinically meaningful varicoceles. For urologists, the WHO's focus on varicocele means that repair remains a key intervention for selected men with infertility and that decision-making should be individualized and aligned with patient values. Furthermore, proper diagnosis requires a clinical (not imaging-based) confirmation of varicocele, and counselling should address realistic expectations regarding semen improvement and timelines for attempting natural conception.

## DISSEMINATION AND IMPLEMENTATION

Effective dissemination and implementation are essential for translating the WHO infertility guideline into meaningful improvements in care. Because infertility services are often fragmented and male evaluation is inconsistently performed, the WHO frames implementation as a health-system strengthening exercise rather than a simple distribution of recommendations.

The guideline is designed for global applicability, and WHO supports its uptake by providing implementation tools, educational materials, and integration pathways linking infertility care to existing reproductive health platforms (Table-7). Countries are encouraged to adapt—rather than merely adopt—the recommendations, ensuring that diagnostic capacity, referral systems, and treatment options reflect local realities. For male infertility, this includes establishing reliable access to semen analysis performed according to the WHO manual; promoting structured history-taking and physical examination in primary care; and creating clear referral pathways for persistent abnormalities, varicocele, or suspected endocrine or genetic causes.

**Table 7:** Key Components of WHO Guideline Implementation for Male and Couple-Based Infertility Care.

Implementation Component	Summary of Application to Male Infertility Care
**Dissemination Strategy**	WHO distributes the guideline through regional offices, workshops, and digital platforms, accompanied by implementation tools and educational materials.
**National Adaptation**	Countries contextualize recommendations based on local diagnostic capacity, surgical expertise, laboratory infrastructure, and financing models. Male evaluation is explicitly included.
**Primary-Level Integration**	Frontline clinicians conduct structured history, focused exam, and semen analysis; address reversible causes; and know when to refer. Ensures men enter the infertility pathway early.
**Referral Pathways**	Clear pathways established for referring men with persistent semen abnormalities, clinical varicocele, endocrine concerns, or suspected genetic/obstructive causes.
**Linkage to Existing WHO Frameworks**	Male infertility services integrated with STI guidelines, tobacco-cessation programs, sexual-health services, and the WHO semen analysis manual.
**Training and Capacity Building**	Urologists and reproductive specialists support education of primary-care providers and help establish minimal andrology laboratory standards.
**Equity and Access**	Implementation aims to reduce disparities by ensuring consistent male evaluation and availability of basic diagnostic and therapeutic services.
**Monitoring and Evaluation**	Countries encouraged to track semen-analysis availability, adherence to diagnostic algorithms, access to varicocele repair, and infertility-treatment outcomes, including pregnancies.

Implementation also requires reinforcing the connection between infertility care and other WHO frameworks, including STI management, tobacco cessation, and sexual-health services. Embedding male infertility within these broader systems improves feasibility and equity by leveraging established infrastructures. At the clinical level, the guideline encourages early engagement of men through prevention counseling, structured diagnostic assessment, and timely referral to specialists. For urologists, this represents an opportunity to lead national and local implementation efforts by training frontline providers, helping develop context-appropriate algorithms, and supporting the establishment of basic andrology laboratory capacity.

Ultimately, effective implementation depends on coordinated action among policymakers, clinicians, educators, and health-system planners. By positioning infertility—male and female—as a core component of reproductive health, the guideline aims to reduce long-standing disparities in access, ensure more consistent evaluation of men, and lay the foundation for equitable expansion of infertility services worldwide.

### Gaps, Research Priorities, and Future Directions

The WHO guideline highlights significant gaps in the evidence supporting male infertility care, many of which limit the strength of recommendations and underscore the need for more rigorous research. While the guideline establishes a global minimum standard, several domains remain insufficiently characterized—scientifically, clinically, and from a public-health perspective.

A major gap concerns the limited evidence supporting therapeutic interventions for male infertility beyond clinical varicocele repair. Despite widespread use of hormonal therapies, including gonadotropins, selective estrogen receptor modulators, aromatase inhibitors, and other empiric medications, high-quality trials powered for pregnancy or live birth remain scarce (55, 85–93). Similarly, the evidence base for antioxidant supplementation is highly heterogeneous, with inconsistent formulations and reliance on surrogate endpoints rather than meaningful reproductive outcomes.

Diagnostic limitations also persist. Conventional semen analysis provides essential baseline information but does not capture molecular or functional sperm attributes such as DNA fragmentation, chromatin architecture, epigenetic signatures, mitochondrial function, or sperm-borne small RNAs. Validation and standardization of these biomarkers are prerequisites for future incorporation into guidelines.

From a public-health perspective, men remain underrepresented in reproductive programs. Better evidence is needed on how to engage men in preconception care, deliver counseling effectively, and integrate male services into primary care, STI programs, and community health settings. Data on access, acceptability, equity, and the psychosocial dimensions of male infertility are also lacking, especially in low- and middle-income countries.

The WHO Evidence-to-Decision framework further highlights priorities related to feasibility, cost-effectiveness, equity, and patient values—domains in which male reproductive health research is particularly thin. Addressing these gaps will be essential for future updates to the guideline and for strengthening global standards of care.

Looking ahead, key research priorities (Table-8) include validating molecular diagnostics, clarifying the paternal contribution to embryo development, expanding access to basic male infertility services, and improving the integration of male care within health systems. These efforts will require coordinated contributions from reproductive biologists, urologists, andrologists, embryologists, public-health experts, and policymakers.

**Table 8:** Priority Research Areas to Strengthen the Evidence Base for Male Infertility Care.

Domain	Key Research Priorities
**Diagnostic Advances**	Validate molecular biomarkers (DNA fragmentation, chromatin structure, epigenetic signatures, sperm RNA cargo). Standardize assays and laboratory methods across settings. Evaluate clinical utility and cost-effectiveness of expanded sperm testing.
**Therapeutic Interventions**	Conduct randomized trials powered for pregnancy and live-birth outcomes for hormonal therapy, empiric medications, and antioxidants. Assess which patient subgroups may benefit from targeted treatments. Compare treatment pathways across resource settings.
**Oxidative Stress & Antioxidants**	Standardize antioxidant formulations and dosing. Link oxidative-stress biomarkers to reproductive outcomes. Clarify potential harms (e.g., reductive stress).
**Varicocele Management**	Evaluate long-term outcomes of different surgical and radiological techniques. Assess effectiveness in subgroups (e.g., borderline semen parameters, elevated sperm DNA fragmentation, various clinical varicocele grades).
**Male Preconception Health**	Identify effective strategies for engaging men in lifestyle modification, tobacco cessation, and STI prevention. Assess effectiveness of interventions according in terms of quality of life and pregnancy outcomes Evaluate implementation models for preconception counseling in diverse settings.
**Public Health & Health Systems**	Characterize barriers to male infertility care globally. Assess equity, acceptability, and feasibility of male-focused services. Develop scalable models for integrating male infertility into primary care and STI programs.
**Psychosocial Dimensions**	Study the mental-health, relational, and social impacts of male infertility. Develop and validate support interventions tailored to men and couples.
**Embryo Development & Paternal Biology**	Investigate associations between paternal health, sperm molecular signatures, early embryogenesis, and pregnancy outcomes. Clarify paternal contributions to miscarriage, implantation failure, and offspring health.

## CONCLUSIONS

The 2025 WHO Guideline for the Prevention, Diagnosis, and Treatment of Infertility marks the first global framework to address both male and female infertility within a unified public-health strategy. By defining minimum standards for prevention, diagnosis, and treatment applicable across all resource settings, the guideline establishes a framework that underscores the importance of male reproductive health. For clinicians working in male infertility, the guideline provides a clear foundation: prevention through modifiable risk reduction, standardized semen analysis following WHO laboratory manual methods, structured history and physical examination, and timely referral to urologists when abnormalities persist. At the same time, the guideline acknowledges its scope limits. It is not a specialty practice document—and is not intended to replace the more detailed andrology-focused guidelines that address condition-specific evaluation and management. It also highlights major evidence gaps, particularly in molecular diagnostics, targeted treatments, oxidative stress, and the broader psychosocial and health-system dimensions of male infertility. Importantly, the guideline reframes male infertility as both a reproductive and a public-health concern. The emphasis on early counseling, lifestyle modification, STI prevention, and couple-based care aligns with growing biological evidence linking paternal health to fertilization, embryo development, and pregnancy outcomes. This perspective encourages a shift from reactive to preventive male reproductive health. Lastly, by integrating feasibility, equity, and scientific rigor, the guideline provides a foundation for clinicians, researchers, and policymakers to build on. Its implementation has the potential to advance reproductive equity, improve diagnostic consistency, and ensure that men worldwide receive timely, structured, and evidence-aligned care. Strengthening the male infertility evidence base will be essential for future updates and for advancing reproductive equity worldwide.

## Data Availability

All data generated or analysed during this study are included in this published article
